# Biomimetic Additive Manufacturing: Engineering Complexity Inspired by Nature’s Simplicity

**DOI:** 10.3390/biomimetics10070453

**Published:** 2025-07-10

**Authors:** Antreas Kantaros, Theodore Ganetsos, Evangelos Pallis, Michail Papoutsidakis

**Affiliations:** Department of Industrial Design and Production Engineering, University of West Attica, 12244 Athens, Greece

**Keywords:** biomimetic additive manufacturing, 4D printing, hierarchical materials, self-assembly, smart materials, bioinspired design, multi-material printing, soft robotics, sustainable manufacturing, structural optimization

## Abstract

Nature’s principles offer design references for additive manufacturing (AM), enabling structures that achieve remarkable efficiency through hierarchical organization rather than material excess. This perspective article proposes a framework for integrating biomimetic principles into AM beyond morphological mimicry, focusing on functional adaptation and sustainability. By emulating biological systems like nacre, spider silk, and bone, AM utilizes traditional geometric replication to embed multifunctionality, responsiveness, and resource efficiency. Recent advances in the fields of 4D printing, soft robotics, and self-morphing systems demonstrate how time-dependent behaviors and environmental adaptability can be engineered through bioinspired material architectures. However, challenges in scalable fabrication, dynamic material programming, and true functional emulation (beyond morphological mimicry) necessitate interdisciplinary collaboration. In this context, the synthesis of biological intelligence with AM technologies offers sustainable, high-performance solutions for aerospace, biomedical, and smart infrastructure applications, once challenges related to material innovation and standardization are overcome.

## 1. Introduction

Biomimetic additive manufacturing (hereafter referred as BAM) is emerging as a contemporary synergetic fabrication technique, combining the foundational principles of biomimicry with the technological flexibility and precision of additive manufacturing (AM) [1]. In this manuscript, the terms “additive manufacturing” (hereafter referred as AM) and “3D printing” are used interchangeably, as they refer to the same family of layer-by-layer fabrication technologies. BAM aims not just to replicate the external appearance of biological entities but to emulate the structural, functional, and material strategies that underlie their remarkable efficiency, adaptability, and resilience [2]. Biological organisms, through millions of years of evolution, have optimized their structures at molecular, micro-, and macroscales to perform complex functions with minimal material consumption and maximal effectiveness [3]. BAM seeks to capture and replicate these embedded design logics within engineered artifacts.

In contrast to traditional design and manufacturing approaches, which often treat geometry, material properties, and function as separate stages of the engineering process, biomimetic strategies promote a deeply integrated philosophy where structure, material, and performance are inseparably linked [4,5,6]. Through the layer-by-layer fabrication paradigm offered by AM technologies, it becomes possible to construct geometrically complex, hierarchical, and multifunctional structures that mirror the organization of biological systems [7]. The capacity to precisely manipulate material composition and spatial arrangement at multiple length scales enables AM to move beyond the constraints of conventional manufacturing toward genuinely bioinspired solutions. While human engineering often equates complexity with sophisticated machinery, multi-material integration, and intricate assembly, nature achieves extraordinary functionality through deceptively simple strategies. Biological systems exhibit complexity not by layering technological interventions, but by optimizing material organization, morphology, and energy flows across multiple scales [8]. For instance, the hierarchical structure of bone or the minimalistic architecture of a spider web achieves outstanding mechanical performance with limited material resources [9,10]. In contrast, human-made designs frequently rely on brute-force material use and mechanical redundancy to attain similar functionality [11,12]. This fundamental distinction reveals an important lesson for BAM [13]: the path to high-performance structures may lie not in increasing manufacturing complexity but in embracing nature’s principles of efficient, purposeful simplicity [14,15,16].

Nature’s strategies—hierarchical structuring, adaptability to environmental stimuli, and multifunctional efficiency—offer a critical blueprint for advancing AM beyond its current capabilities. Hierarchical structuring, observed in systems such as nacre or plant stems, confers remarkable combinations of strength, toughness, and lightness [17]. Adaptability, seen in dynamic systems like pinecones or octopus’ arms, informs the development of responsive 4D-printed materials capable of evolving shape or function over time [18]. Efficiency in material usage, prevalent across the biological kingdom, suggests paths toward more sustainable manufacturing practices by minimizing waste and optimizing load-bearing structures [19]. By studying and applying these principles, ΒAΜ can move beyond static reproduction of natural forms toward dynamic, resilient, and resource-efficient systems that emulate the profound intelligence embedded in biological design [20]. These principles offer a potential blueprint for advancing additive manufacturing beyond its current capabilities. This is particularly timely considering the growing demands for sustainable production, material efficiency, and climate-resilient design inter- and intra-industries such as aerospace, healthcare, and construction.

The purpose of this perspective article is to critically examine how engineering complexity can be achieved through nature’s simplicity by leveraging biomimetic principles within AM frameworks. Rather than providing an exhaustive review of existing studies, this article aims to articulate conceptual pathways for future innovation, highlight current technological gaps, and propose a strategic vision for the next generation of BAM research and applications. Special emphasis is placed on the transition from mimicking biological shapes to embedding biological functions and adaptive behaviors into printed systems. Through this discussion, a gradual structural change is discussed: designing for complexity not through the addition of components and materials, but through the intelligent orchestration of simple, nature-inspired principles that provide higher degrees of performance, sustainability, and adaptability in engineered systems.

While the term “nature’s simplicity” is used in the title, it should be understood in the context of design elegance rather than structural reduction. Biological systems are indeed characterized by high levels of complexity, from nanoscale molecular assemblies to macroscale architectures. However, nature often achieves functional richness not through excess, but through efficient use of materials, hierarchical structuring, and self-organization. The phrase is thus intended to reflect the minimalist and purposeful strategies that underpin biological efficiency—principles that can inspire the development of high-performance, resource-conscious engineering solutions.

This manuscript is structured to reflect a logical progression from natural principles to engineering applications. Section 2 presents biological examples of structural efficiency and simplicity across molecular, microscale, and macroscale levels, serving as a foundation for design inspiration. Section 3 discusses the translation of these biomimetic strategies into AM, focusing on the integration of geometry, material properties, and functionality. Section 4 highlights recent advances in dynamic and responsive systems—including 4D printing, soft robotics, and self-morphing structures—that exemplify the potential of ΒAΜ. Section 5 addresses current challenges related to materials, scalability, and functional integration. Section 6 outlines emerging directions and future perspectives, including developments in machine learning, hybrid bio-systems, and sustainability. Finally, Section 7 provides concluding remarks and identifies opportunities for further interdisciplinary research and application.

## 2. Nature’s Simplicity: Examples at Different Scales

### 2.1. Molecular Scale: Self-Assembly and Minimal Energy Structures

At the molecular level, nature achieves remarkable functionality through the self-organization of basic building blocks under thermodynamic and kinetic principles. Structures such as DNA origami, protein folding, and lipid bilayers exemplify minimal energy configurations that produce highly stable and functional forms [21]. DNA, for instance, self-assembles into intricate three-dimensional architectures by exploiting specific base-pairing rules, allowing for the creation of complex nanoscale shapes with precision and predictability [22,23]. Similarly, proteins fold spontaneously into functional conformations, minimizing free energy while maximizing biological efficacy [24,25]. These processes highlight an essential strategy: complexity and specificity in natural systems often emerge not from external design interventions, but from the inherent informational content embedded within simple molecular subunits and their energetic interactions [26].

**Figure 1:**
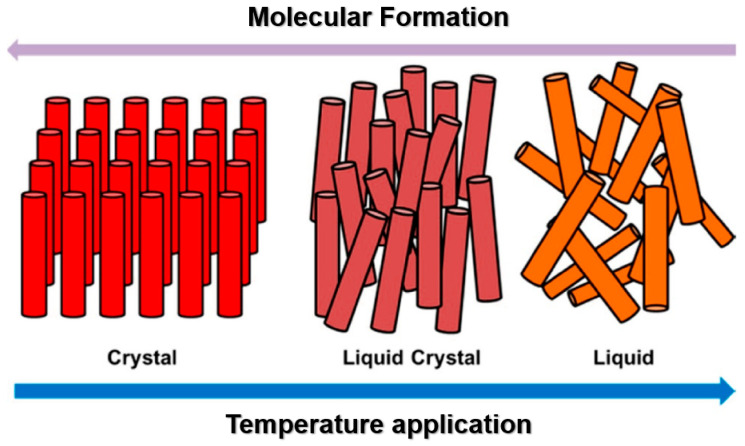
LCEs during their phase transformation with the application of external stimuli [30].

For ΒAΜ, the molecular scale offers critical inspiration regarding how self-assembly principles might be integrated into fabrication processes [27]. Incorporating self-assembly mechanisms into AM workflows could enable the construction of complex architectures without the need for intricate external manipulation, reducing energy input and fabrication time [28]. Furthermore, minimal energy design strategies at the molecular level suggest pathways for creating responsive, self-healing, or adaptive materials that autonomously maintain or recover functionality, much like biological systems [29]. Understanding and incorporating these molecular-level strategies into the aforementioned processes may redefine what is possible in next-generation AM, shifting the emphasis from laborious construction toward intelligent material programming. Liquid Crystal Elastomers (hereafter referred as LCEs), which exhibit phase transitions in reaction to external stimuli, provide an illustration of this molecular-level approach, demonstrating how fundamental material-level principles can be harnessed to create complex, adaptive behavior. Such materials illustrate the fundamental biomimetic concept of attaining function not by increased complexity but by the smart design of natural material qualities. In this context, Figure 1 depicts Liquid Crystal Elastomers (LCEs) during their phase transformation with the application of external stimuli [30].

While molecular systems depict how self-assembly and minimal energy configurations can achieve the desired functionality, the microscale introduces hierarchical structuring based on these principles to enable load-bearing and multifunctional performance.

### 2.2. Microscale: Hierarchical Yet Minimalistic Structures

At the microscale, nature produces highly optimized structures that combine hierarchical organization with material economy [31]. Nacre, or mother-of-pearl, exhibits a “brick-and-mortar” microarchitecture composed of aragonite platelets and organic layers and exhibits exceptional toughness through crack deflection and energy dissipation, despite being made from relatively weak constituent materials [32,33]. The lotus leaf, with its micro- and nanostructured surface topography, achieves superhydrophobicity and self-cleaning properties without the use of chemical treatments [34,35,36]. Likewise, spider silk demonstrates a microscale organization that balances lightweight construction with outstanding tensile strength and elasticity, arising from precise molecular arrangement within fibrils [37]. These examples reveal how microscale structuring achieves superior performance with minimal material input, guided by principles of hierarchical ordering and functionality maximization [38].

For ΒAΜ, microscale designs offer a blueprint for achieving multifunctional, high-performance systems without the need for excessive material complexity [39]. Emerging high-resolution printing techniques, such as two-photon polymerization [40] and advanced electrospinning [41], are beginning to approach the ability to replicate such fine hierarchical features [42,43]. However, realizing true biomimetic efficiency requires not only duplicating these microstructures but understanding their functional interdependencies and scaling relationships. By designing AM processes that incorporate microscale features strategically, it is possible to create surfaces, composites, and metamaterials that mimic the multifunctionality of natural systems [44], enabling innovations across sectors such as biomedical devices [45], wearable sensors [46], and smart surfaces [47]. Based on microscale hierarchical designs, the macroscale level extends these concepts to achieve adaptive, load-optimized forms meeting the imposed environmental and mechanical demands.

### 2.3. Macroscale: Load-Optimized, Adaptive Forms

At the macroscale, biological systems exemplify load-optimized and environmentally responsive architectures. Trees, for example, optimize their growth patterns to distribute mechanical stresses evenly, resulting in forms that withstand dynamic loading from wind and gravity with minimal material use [48,49]. Bone, a highly adaptive material, remodels itself in response to mechanical stimuli, maintaining strength while minimizing mass through a porous, hierarchical structure [50,51,52]. Shells, such as those of mollusks and turtles, combine curvature, gradient material composition, and structural reinforcement to provide formidable protection without excessive weight [53,54]. These macrostructures embody nature’s capacity to achieve functional resilience through the strategic deployment of material, geometry, and adaptive capability [55].

**Figure 2:**
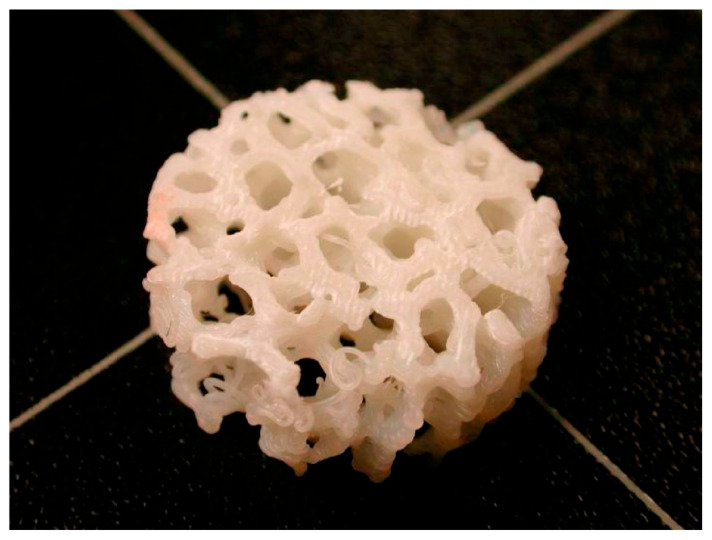
Biomimetic additive manufacturing of bone scaffold structure [61].

ΒAΜ at the macroscale aims to incorporate these strategies into engineered systems by developing structures that can optimize load distribution, adapt to changing conditions, or self-repair over time. Advances in large-scale AM, particularly in topology optimization algorithms coupled with 3D printing, are beginning to realize designs that mimic the lightweight strength of bones or the protective efficiency of shells [56,57,58]. Furthermore, programmable materials [59] and 4D printing open possibilities for constructing adaptive macrostructures capable of evolving in response to environmental stimuli [60], much like natural organisms [61]. By learning from nature’s macroscale simplicity—where form follows function—engineered products can achieve unprecedented levels of efficiency, adaptability, and sustainability. In this context, Figure 2 depicts a bone scaffold structure, fabricated with AM methods, designed to fulfill biomimicry criteria. At the macroscale level, bone scaffolds fabricated via additive manufacturing techniques depict how hierarchical structuring can achieve both mechanical and biological functions with minimal material use. This example highlights how biomimetic principles can lead to the creation of load-optimized, adaptive designs that go beyond simple geometric replication to achieve multifunctional performance.

The complexity of biological systems is not only spatial and functional but also evolutionary. Constructs that have persisted over vast evolutionary timescales—such as shells, corals, and bone—often exhibit highly optimized architectures shaped by millions of years of selective pressure. These systems, despite their ancient origin, frequently display advanced characteristics such as gradient material distribution, self-adaptation, and minimal material use, all embedded within a hierarchical framework. As highlighted by Ehrlich [62], even relatively simple-seeming biological structures can pose substantial challenges for AM due to their intricate internal organization. Incorporating the evolutionary maturity of a structure provides valuable context for understanding both the sophistication of natural design and the limitations of current fabrication technologies in replicating such complexity.

Together, these three scales—molecular, microscale, and macroscale—demonstrate how nature integrates simplicity and efficiency across hierarchical levels to achieve peak performance. Understanding and incorporating these multiscale strategies into AM leads to the development of next-generation biomimetic systems that combine adaptability, multifunctionality, and sustainability.

## 3. Translating Simplicity into Additive Manufacturing

ΒAΜ seeks to harness the fundamental principles of nature’s efficiency, striving to replicate the geometric, material, and functional characteristics observed in biological systems. Through the utilization of advanced AM (AM) technologies, there is an increasing effort to emulate the intricate designs that have evolved in nature, such as complex lattice structures and gyroid geometries, which optimize structural performance while minimizing material consumption. Moreover, the development of materials that exhibit hierarchical gradients, as well as multifunctional properties such as self-healing and responsiveness, represents a critical avenue of research within BAM. Despite these advancements, numerous challenges persist in fully translating the simplicity and sophistication of nature into engineered systems, particularly in relation to the limitations of current materials, the resolution capabilities of AM processes, and the integration of complex, adaptive functionalities within fabricated structures. This chapter critically examines the ongoing efforts to translate nature-inspired designs into the sector of AM and explores the key barriers that must be overcome to achieve their full realization.

It is important to distinguish between biomimetic constructs inspired by biological systems and direct imitations of complex biological organs or tissues. While AM techniques have enabled the printing of structures that resemble biological forms, these printed imitations do not replicate the full multiscale functionality or dynamic responsiveness of living systems. Our perspective focuses on biomimetic constructs that integrate selected principles from biology—such as hierarchical organization, adaptive morphology, or material efficiency—for extracorporeal engineering applications. These include smart materials, structural components, or devices whose performance is enhanced by bioinspired design, rather than by mimicking organ-level biological function.

### 3.1. Geometry, Material, and Function: Towards Integrating Bioinspired Designs in AM

One of the central elements of ΒAΜ lies in the integration of geometry, material properties, and functionality within a single design framework [63]. Traditional manufacturing approaches often treat these elements separately: geometry is defined first, followed by material selection, and finally the assignment of functional roles. In contrast, nature’s systems inherently merge these aspects, where the form of a structure is intrinsically linked to its function and material composition. This holistic approach enables the construction of highly optimized systems that perform complex tasks with minimal material expenditure. AM technologies, with their capacity for precise, layer-by-layer deposition of materials at multiple scales, provide an ideal platform for replicating such integrated designs. For instance, lattice structures that mimic the trabecular bone’s lightweight yet strong architecture, or gyroid geometries, which resemble coralline structures, have been successfully produced using advanced 3D printing techniques [64]. These bioinspired geometries optimize structural performance, reducing material consumption while enhancing mechanical efficiency. Moreover, bioinspired designs in AM require a deeper understanding of how material properties must be tailored to function seamlessly within the geometry, creating a synergy that maximizes performance [65]. Therefore, achieving this level of integration demands not just the replication of natural forms but also the strategic manipulation of materials and geometries to meet specific functional requirements, shifting from esthetic mimicry to functional biomimetic engineering [66].

In nature, the geometry of a structure is not arbitrary but is the result of millions of years of evolutionary optimization aimed at enhancing function while minimizing resource use. For example, the honeycomb structure in beehives maximizes strength and efficiency with minimal material by utilizing a hexagonal geometry that provides structural stability under compression while reducing the material cost [67,68]. Similarly, in plants, branching patterns exhibit fractal-like structures, enabling efficient light capture and material distribution [69,70]. In AM, replicating such geometries involves leveraging the precision of 3D printing technologies to construct complex forms that align with the principles of material optimization [71,72]. Lattice structures, for instance, replicate the microarchitecture of bones [73], offering high strength-to-weight ratios [74] while minimizing material waste [75,76,77]. Gyroid structures, with their intricate yet stable design patterns exhibit multifunctional properties such as lightweight strength, permeability, and even thermal regulation [78,79]. By incorporating these bioinspired geometries into additively manufactured items, it becomes possible to create materials and structures that closely mimic the optimized forms found in nature, potentially improving both the efficiency and sustainability of engineered systems.

The second pillar of ΒAΜ has to do with the development of materials that emulate the properties of biological systems, with an emphasis on versatility, adaptability, and multifunctionality. Biological materials, such as bone, muscle tissue, and spider silk, exhibit remarkable properties that arise from their hierarchical structures, which enable them to perform multiple functions while maintaining high strength and minimal material consumption. Bone, for example, is composed of mineralized collagen fibers arranged in a hierarchical fashion that enables it to bear substantial loads without becoming brittle [80,81]. Similarly, spider silk combines extraordinary tensile strength with elasticity, enabling it to absorb high amounts of energy. In the sector of AM, the challenge is to develop materials that replicate these qualities, often using gradient composites or metamaterials [82,83]. Gradient composites vary their material properties across different layers, mimicking the functionally graded structures found in nature, such as the transition from soft to hard materials in seashells [84,85]. Metamaterials, engineered materials with properties not found in nature, can be tailored to achieve unique characteristics such as negative thermal expansion or enhanced mechanical resilience [86,87]. However, the complexity of replicating these biological materials in the form of printable raw materials presents significant challenges, particularly in terms of scalability, durability, and the ability to respond adaptively to environmental stimuli [88]. Advances in smart materials, such as shape-memory polymers and responsive hydrogels, hold promise in bridging the gap between nature’s material capabilities and their AM counterparts.

Beyond geometry and material composition, the third critical element of ΒAΜ is the integration of functional capabilities, such as responsiveness and adaptability, that are inherent in natural systems [89]. Biological structures are often designed to respond dynamically to their environment, adjusting their properties or shape in response to external stimuli. For example, muscles can contract in response to electrical signals, while tree branches bend to adapt to wind conditions, ensuring the survival and optimal performance of the organism. In AM, integrating such functional behaviors presents a significant challenge but also an exciting opportunity to advance the field [90]. The development of 4D printing, which incorporates time as a variable through the use of shape-memory materials or stimuli-responsive polymers, is a key step toward creating systems that can change their form or functionality in response to environmental factors such as temperature, light, or moisture [91]. This concept of adaptability is evident in the potential for self-healing materials, which can recover from damage autonomously, mimicking biological healing processes [92]. However, achieving such functionality requires a comprehensive understanding of how to encode these behaviors into the materials and structures produced by AM technologies [93]. As this subsector progresses, the ability to embed biological-like functions—such as adaptability, self-healing, and environmental responsiveness—into 3D-printed materials will be crucial for advancing the next generation of bioinspired, high-performance systems.

### 3.2. Challenges: Material Limitations, Printing Resolution, and Integration of Functionality

The full realization of ΒAΜ involves not only replicating the geometric complexity found in biological systems but also overcoming a series of inherent challenges related to material limitations, printing resolution, and the integration of functionality. While current AM technologies have made significant progress in mimicking the shapes and structures of natural organisms, a substantial gap persists between the capabilities of existing 3D printing methods and the sophisticated materials and dynamic functionalities that biological systems exhibit. Biological materials are often multifunctional, adaptable, and responsive, capable of responding to environmental stimuli or undergoing self-repair in real time [94]. Achieving this level of complexity and adaptability in 3D-printed systems requires addressing several key obstacles: the development of new materials with tunable properties, advancements in printing resolution to capture fine-scale features, and the integration of dynamic functionalities into printed objects [95]. This subchapter delves into these challenges, exploring the current limitations and the research efforts aimed at overcoming them to bring the promise of ΒAΜ closer to reality.

One of the most significant challenges in the field of ΒAΜ lies in overcoming the material limitations that currently do not allow the full realization of bioinspired designs. Biological systems exhibit remarkable material properties, such as exceptional strength-to-weight ratios, resilience, and adaptability, which are the result of millions of years of evolutionary optimization [95]. For instance, materials like spider silk and bone are capable of absorbing significant amounts of stress and strain without failure, thanks to their hierarchical structures and the sophisticated interplay of natural polymers and minerals. Replicating these sophisticated material behaviors in the context of AM remains a substantial challenge. While the current materials available for 3D printing, such as thermoplastics, resins, and metals, offer certain advantages, they often fall short in mimicking the versatility and multifunctionality of biological materials [96]. Traditional materials tend to be static, with fixed properties that do not allow for adaptive responses or dynamic changes in functionality over time. The absence of materials that can emulate the complex, tunable properties seen in nature—such as self-healing, shape memory, or biocompatibility—limits the scope of potential applications for BAM. Efforts are underway to develop new materials, including composites, metamaterials, and bio-based polymers, that combine the desired mechanical properties with enhanced adaptability and responsiveness [97]. However, achieving this material versatility remains a significant research frontier. Until advanced materials that can exhibit the same level of complexity and multifunctionality as biological materials are developed, potential ΒAΜ will remain constrained.

Another formidable challenge in ΒAΜ is the limitation of printing resolution, which impacts the ability to replicate the fine-scale features found in biological systems [98]. In nature, structures are organized at multiple scales, from the molecular to the macroscale, with each level of organization contributing to the overall functionality of the system. As mentioned prior in the text, the nanoscale features of a lotus leaf surface contribute to its superhydrophobicity, while the microscale structure of spider silk imparts extraordinary tensile strength [99]. To replicate such complex hierarchies using AM, the printing resolution must be fine enough to accurately reproduce these micro- and nanoscale features. While high-resolution 3D printing technologies, such as two-photon polymerization, direct laser writing, and micro-extrusion, have made significant strides in this area, they still face substantial limitations in terms of scalability, speed, and material compatibility [100]. Most current printing technologies, especially those used in large-scale AM, are limited to resolutions that fall within the millimeter or micrometer range, which is insufficient to faithfully reproduce the intricate details of biological microstructures [101]. Furthermore, the integration of fine-scale features into larger, more complex geometries presents challenges in ensuring that the material properties and functionality of the printed object are maintained across different scales. The inability to seamlessly bridge the gap between the nanoscale and macroscale hampers the ability to achieve true biomimetic designs that function as naturally as biological systems.

Apart from material limitations and printing resolution, one of the most significant challenges in ΒAΜ is the integration of functionality into printed structures. While traditional manufacturing processes focus primarily on creating passive components with predetermined properties, biomimetic manufacturing seeks to replicate not only the form but also the dynamic functionality of biological systems. Natural organisms are capable of adapting to their environment, responding to stimuli, and performing complex functions such as self-healing, regeneration, and environmental sensing [101,102,103]. For example, bones remodel themselves in response to mechanical stress, and plants exhibit phototropic growth in response to light [104,105]. The challenge for AM is to integrate these dynamic, adaptive behaviors into 3D-printed structures. This requires the development of advanced materials that can change their shape, mechanical properties, or even chemical composition in response to external stimuli [106]. While the field of 4D printing, which involves the use of shape-memory polymers, hydrogels, and other stimuli-responsive materials, has made sufficient progress, significant hurdles remain in terms of achieving consistent performance, long-term stability, and scalability of these materials [107,108,109]. Additionally, embedding functional behaviors such as self-healing or environmental responsiveness requires a deep understanding of how these processes operate at the molecular and structural levels [110]. Designing and manufacturing systems that can replicate these capabilities at scale—while maintaining the structural integrity and performance of the final product—is a complex, multidisciplinary challenge [111]. The following table (Table 1) summarizes the key aspects, bioinspired examples, AM approaches, and current challenges associated with integrating geometry, material properties, and functionality in ΒAΜ.

## 4. Complexity Through Simplicity: Novel Capabilities in BAM

The continued evolution of ΒAΜ marks a structural change from mimicking static biological forms toward embedding dynamic, adaptive behaviors into synthetic systems. At the forefront of this transition is the exploration of design strategies that draw inspiration not solely from biological structures, but from the processes and temporal patterns that describe how living systems interact with their environments. As such, BAM is increasingly intersecting with emerging fields like programmable matter, embodied intelligence, and active morphogenesis, leading to systems capable of transformation, self-regulation, and distributed responsiveness. This section focuses on three major technological domains—4D printing, soft robotics, and self-morphing structures—that exemplify this shift toward time-sensitive and behaviorally enriched fabrication paradigms. Collectively, these approaches open new avenues for designing systems that do not passively reflect biology, but exhibit lifelike adaptability, autonomy, and context-aware performance across a range of applications.

### 4.1. Four-Dimensional Printing and Programmable Materials: Dynamic Behaviors Inspired by Plants or Skin

Four-dimensional printing, defined by the incorporation of time-dependent transformations into fabricated objects, extends the capabilities of AM by allowing structures to evolve post-fabrication in response to predefined stimuli. While earlier iterations of BAM focused on replicating complex geometries, 4D printing introduces a kinetic dimension that enables components to undergo controlled transformations after printing, thereby introducing adaptability without external actuation systems.

What distinguishes 4D printing in the biomimetic context is its capacity to encode behavioral logic into material systems. Inspired by phenomena such as nastic movements in plants [112] or thermoregulatory behavior in skin [113], 4D-printed components can be pre-programmed to fold, unfold, expand, or contract in response to specific environmental cues [114]. Unlike conventional actuation systems that rely on centralized control, these transformations are inherently embedded in the material architecture and triggered by ambient changes, resulting in decentralized and energy-efficient adaptations.

Recent advances have demonstrated the feasibility of integrating spatial and temporal gradients into single printed objects using multi-material extrusion, anisotropic layering, or voxel-wise material programming [115]. These methods have enabled the fabrication of devices that autonomously transition between states, opening possibilities for use in minimally invasive surgical tools, transient biomedical scaffolds, and deployable habitat systems [116]. As the field matures, future research is expected to focus on the refinement of spatiotemporal control, reversibility of deformation, and lifecycle predictability of programmable materials under prolonged exposure to complex environmental conditions.

### 4.2. Soft Robotics: Bioinspired Movement Without Rigid Components

Soft robotics, inspired by the biomechanical strategies of soft-bodied organisms, represents an evolution in robotics by eschewing rigid kinematic chains in favor of compliant, deformable, and continuously actuated bodies [117]. Within BAM, this shift aligns with the broader ambition to fabricate systems that exhibit embedded intelligence and adaptability through morphological computation.

Rather than relying on centralized electronics or hard mechanical joints, soft robotic systems distribute function across their material continuum, exploiting elasticity, viscosity, and strain distribution to achieve locomotion or manipulation [118]. This makes them especially suited for tasks that require conformity to unstructured environments, high degrees of freedom, or delicate interaction with biological tissues.

The integration of BAM into soft robotics introduces novel capabilities in customizing the mechanical response of actuators through spatially graded stiffness, internal channels, or embedded sensing elements—all achievable in a single manufacturing workflow [119]. Applications include biointegrated devices for assistive movement, shape-shifting end-effectors for precision agriculture, and wearable robots that synchronize with the user’s motion intent [120,121]. Unlike traditional fabrication, BAM allows for tailoring robot morphology to specific performance envelopes, offering unprecedented control over how form contributes to function.

Key research directions now focus on extending autonomy in soft robots via energy harvesting skins, proprioceptive feedback systems, and decentralized control logic, all of which can benefit from the integration of BAM processes capable of embedding such systems during fabrication rather than through post-processing [122,123].

### 4.3. Self-Morphing Structures: From Simple Stimuli to Complex Shape Transformations

Self-morphing structures encapsulate a class of systems whose final configuration is not fully realized at the moment of fabrication but emerges in response to environmental conditions or internal programming [124]. Rooted in the principles of morphogenetic design and origami/kirigami mechanics, these structures offer a compelling framework for BAM to create functionally graded, spatially responsive artifacts with minimal mechanical input [125].

Unlike 4D printing, which typically relies on predictable stimulus–response cycles, self-morphing structures may exhibit irreversible or partially reversible transformations that are designed to occur at critical moments in a system’s lifecycle—such as deployment, failure prevention, or task-specific reconfiguration [126]. Inspiration is drawn from diverse natural phenomena, including seed dispersal mechanisms, tissue folding in embryogenesis, or hygroscopic plant structures that actuate upon hydration [127].

BAM facilitates the realization of such mechanisms by enabling multiscale patterning of expansion coefficients, anisotropic strain distributions, or built-in geometric instabilities. Recent work has demonstrated the viability of printing flat precursors that morph into 3D load-bearing forms upon exposure to environmental triggers, such as humidity, temperature, or pH variation [128]. These systems have potential applications in space-deployable components, adaptive shading systems in architecture, or biomedical devices that activate upon implantation [129].

A major research frontier lies in the codification of design rules that link stimuli, material configuration, and resulting morphologies [130]. This entails the use of predictive computational models, coupled with real-time environmental simulations, to inform material and geometric choices during the design phase [131]. As self-morphing technologies evolve, BAM offers a targeted, suited platform for fabricating high-performance, autonomous transformations embedded in the very structure of the object itself. Table 2 summarizes the aforementioned emerging frontiers in biomimetic additive manufacturing (BAM).

## 5. Challenges and Gaps

The advancement of BAM depends on overcoming several key scientific and technological barriers that currently limit its broader application. One of the most significant limitations lies in the capacity to develop synthetic materials that replicate the multifaceted and dynamic properties exhibited by biological systems [132]. While a growing array of engineered materials—including shape-memory polymers, hydrogels, and bio-derived composites—have been integrated into BAM processes, these substances remain insufficient in emulating the sophisticated, multifunctional performance of their natural analogues [132]. In biological contexts, materials do not merely fulfill structural roles but frequently act as active, adaptive components capable of self-regulation, environmental responsiveness, and functional evolution. The replication of such attributes necessitates not only the refinement of existing material classes but also the conception and synthesis of novel materials whose behavior is guided by biological principles.

Achieving this objective requires a radical alteration in the way materials are conceptualized within AM frameworks. Rather than being treated as static media to be shaped, materials in BAM must be designed as programmable systems with inherent capacities for transformation and interaction with their environment. Such behavior, commonly observed in natural systems—where materials respond to stimuli such as mechanical stress, moisture, temperature, or light—presents a formidable challenge to current material science [133]. Engineering materials with similar capabilities entails interdisciplinary knowledge spanning molecular dynamics, responsive chemistry, and biofunctional mechanics [134]. The development of such bioequivalent smart materials remains in its infancy and represents a key research frontier essential for enabling BAM to replicate not only the form but also the autonomous functional behaviors observed in living systems.

In parallel with material challenges, issues related to scalability and fidelity pose considerable obstacles to the practical deployment of BAM [135]. Biological systems exhibit hierarchical organization across multiple spatial scales, with functionalities often emerging from the interplay between micro- and macrostructural features [136,137]. While AM enables high-resolution control over geometry, replicating this multiscale integration with consistent precision remains technologically constrained. The transition from small-scale prototypes to full-scale, application-ready structures frequently results in diminished structural fidelity, heterogeneity in material properties, and increased susceptibility to fabrication errors [138]. These limitations are particularly acute when reproducing bioinspired architectures that rely on spatially graded properties or complex topologies, which are difficult to maintain uniformly at larger volumes.

Addressing scalability in BAM requires not merely incremental improvements in existing manufacturing techniques, but a broader reconceptualization of fabrication strategies [139]. Approaches such as hierarchical assembly, modular construction, and process hybridization may offer viable pathways to bridging the gap between microscale fidelity and macroscale applicability. Furthermore, the integration of algorithmically driven design methodologies and real-time feedback systems could facilitate the adaptive calibration of process parameters, enhancing reproducibility and enabling responsive fabrication workflows [140]. These innovations are essential for extending the utility of BAM into domains such as aerospace, biomedical engineering, and architecture, where large-scale, structurally efficient, and functionally complex systems are in high demand.

A final, yet equally important, barrier to progress in BAM lies in the persistent di-vergence between esthetic and functional mimicry. While AM has demonstrated considerable success in reproducing the visual and geometric characteristics of natural forms, the replication of underlying biological functions remains largely unfulfilled. This limitation is not trivial. In nature, structural forms arise from evolutionary pressures that optimize for multiple, often competing, performance criteria, including mechanical robustness, adaptability, and responsiveness. Replicating only the outward appearance of these forms without embedding their functional logic undermines the potential impact of BAM and restricts its applicability in scenarios demanding intelligent, adaptive behavior.

Closing the gap between esthetic and functional biomimicry necessitates a deeper integration of biological insight into the design and fabrication processes. This includes the translation of biological mechanisms—such as self-healing, environmental sensing, and motion regulation—into engineering principles that inform both material selection and structural design [141]. Achieving this level of biofunctionality requires close interdisciplinary collaboration among material scientists, biologists, and engineers, as well as the deployment of advanced computational tools capable of simulating and optimizing complex, bioinspired systems [142,143]. Ultimately, it is through this convergence of disciplines and methodologies that BAM can move beyond surface-level imitation and realize its full potential as a platform for engineering adaptive, multifunctional systems.

Despite significant advancements, current AM technologies still fall short of meeting the multifaceted demands of biomimetic applications. For instance, printing resolution remains a limiting factor: while microscale features are essential to replicate biological structures such as trabecular bone or lotus leaf surfaces, most commercial AM systems operate within the 50–200 µm range, which is insufficient for nanoscale precision required in certain BAM use cases. Similarly, material diversity and functional performance are limited—most AM processes rely on thermoplastics, photopolymers, or metals with fixed mechanical and thermal properties, lacking the adaptability, self-healing ability, or multifunctionality observed in biological materials. BAM thus represents not only a conceptual extension of AM, but a necessary evolution to meet these emerging needs, requiring new printable materials with graded or stimuli-responsive behavior and resolution enhancements capable of spanning multiscale structural integration. These needs highlight the importance of interdisciplinary innovation at the interface of material science, bioengineering, and AM technology.

In summary, the future progression of ΒAΜ depends on addressing three interrelated challenges: the development of biologically analogous smart materials, the realization of scalable and high-fidelity fabrication processes, and the achievement of true functional mimicry beyond visual resemblance. These barriers are inherently interdisciplinary in nature, requiring sustained innovation at the intersection of materials science, AM technology, and biological understanding. Overcoming them will be of paramount importance in enabling BAM to deliver on its promise of producing engineered systems that mirror the efficiency, adaptability, and complexity of nature. Table 3 summarizes the aforementioned key challenges in biomimetic additive manufacturing (BAM) and current solutions.

## 6. Future Perspectives

Biomimetic additive manufacturing (BAM) is transitioning from an emerging research focus into a foundational strategy for developing next-generation engineering solutions. Its future lies in synergizing nature-inspired principles with technological advances that extend far beyond material replication. This chapter envisions the potential future perspectives in BAM, reflecting the integration of machine learning, biohybrid systems, and sustainability ethics into a holistic framework for innovation.

One of the most important developments shaping the trajectory of BAM is the integration of artificial intelligence (AI)—particularly machine learning (ML) and generative design algorithms—into the design and fabrication pipeline [144]. These tools enable the automatic discovery of optimal structures by learning from vast datasets of natural and engineered systems. Rather than depending solely on human intuition, ML models can predict performance outcomes based on complex geometric, mechanical, and biological parameters [145,146]. Generative design, in turn, iterates through millions of design permutations to identify geometries that mirror the structural efficiencies observed in natural forms. Together, these tools accelerate innovation, reduce trial-and-error prototyping, and promote resource-efficient structures with superior functional performance [147,148,149].

Parallel to algorithmic design evolution is the emergence of living materials and hybrid systems, a domain that merges biology with engineering at an unprecedented scale. Biohybrid printing—where living cells are combined with synthetic matrices—introduces the possibility of creating systems capable of growth, self-repair, and environmental responsiveness [150]. In healthcare, this may result in patient-specific bioactive implants or tissue scaffolds that dynamically integrate with the body [151]. In environmental engineering, biohybrids could lead to responsive ecosystems or regenerative urban materials [152]. These advances bridge the gap between animate and inanimate systems, advancing BAM capabilities toward a future where printed matter behaves less like machinery and more like organisms.

Yet, these breakthroughs bring with them a set of pressing ethical, ecological, and sustainability challenges. The use of living organisms and biologically active materials in manufacturing necessitates a reevaluation of safety protocols, regulatory standards, and ecological impact [153,154]. The lifecycle of BAM products—spanning material sourcing, energy-intensive printing processes, and end-of-life disposal—must be critically assessed [155]. There is a risk that innovation could outpace responsible implementation, especially in domains where ecological stability or human health is at stake [156]. Consequently, the future of BAM must be shaped not only by technological possibility but also by robust ethical inquiry and environmental stewardship. To transform these advances into responsible innovation, researchers should prioritize the development of bio-based, recyclable materials; industrial sectors must invest in scaling sustainable manufacturing processes; and policymakers should work toward planning clear regulatory frameworks that combine innovation with environmental and societal considerations.

The field will further benefit from multi-level standardization frameworks [157,158]. While BAM currently excels in bespoke and experimental production, its industrial-scale adoption will require consistent guidelines for biomimicry validation [158,159], material behavior benchmarking [160,161], and long-term performance evaluation [162,163,164]. Such frameworks should be developed in coordination with regulatory agencies, academic institutions, and industry partners to ensure scalable, safe, and repeatable applications across sectors [165,166].

Finally, interdisciplinary convergence is considered of paramount importance for the successful application and evolution of the technique [167,168,169]. The complexity of BAM applications—from soft robotics [170,171] and 4D-printed implants [172,173] to climate-responsive building skins [174,175]—demands collaboration across fields including synthetic biology, computational design, materials chemistry, architecture, and biomedical engineering. Equipping future researchers with hybrid competencies and promoting institutional structures that support cross-disciplinary projects will be essential to fully realize the potential of BAM.

In essence, the future of biomimetic additive manufacturing will be defined not only by how well nature’s forms are replicated, but by how ethically, sustainably, and intelligently its principles are integrated. BAM’s potential is defined in fabricating systems that, like biological counterparts, embody efficiency, adaptability, and resilience—while also reflecting the responsibility and foresight required in designing with and for life. Table 4 summarizes the aforementioned future perspectives in biomimetic additive manufacturing (BAM).

## 7. Conclusions

ΒAΜ represents an evolution in the way researchers approach engineering design, reconsidering traditional methods of complexity and innovation. Nature’s inherent capacity for self-organization and multiscale optimization offers a blueprint for creating systems that are not only functional but also efficient and adaptable. The simplicity of biological systems—ranging from the molecular self-assembly of DNA to the macroscale adaptive structures found in trees, bones, and shells—demonstrates that complexity does not necessitate a high degree of material or structural intricacy. Instead, nature achieves its functional purposes through efficient, minimalistic designs that utilize hierarchical organization and material optimization, principles that have the potential to structurally alter engineering disciplines. By utilizing these principles, BAM leads to the development of highly optimized, multifunctional materials and structures that can outperform traditional designs in terms of strength, adaptability, and sustainability.

Thus, it is imperative that the field of AM evolves towards a deeper integration of bioinspired efficiency. Current technological advancements, while of paramount importance, have not greatly evolved towards bioinspired design philosophies, which should be fully integrated into the AM process. Moving forward, it is crucial to reassess AM strategies to prioritize not only the replication of natural forms but the functional principles that underlie their success. This reassessment should involve a closer examination of the molecular, microscale, and macroscale strategies employed by nature to achieve performance through simplicity. By changing the focus from complex human-imposed designs to the natural efficiencies embedded in biological systems, it will be possible to create artifacts that are not only more sustainable but also far more adaptable and responsive to the high demands of modern engineering.

Drawing on nature’s time-tested techniques to attain functionality, efficiency, and adaptabibility across scales, biomimetic additive manufacturing (BAM) provides new abilities for the bioengineering sector. As the field advances, it is crucial to rethink AM strategies not only as a technique for replicating natural forms, but as a platform for embedding the functional intelligence inherent in biological systems. By refocusing from complex, human-imposed designs to the natural efficiencies embedded in living systems, engineered artifacts can be created that are not only more sustainable, but also more resilient, adaptive, and ecologically responsible. Ultimately, the evolution of BAM will depend on the ability to harmonize technological innovation with the principles of biological design, leading to novel sustainable and intelligent materials and structures.

## Figures and Tables

**Table 1:** Key elements and challenges in translating biomimetic principles into additive manufacturing.

Aspect	Nature-Inspired Model	Additive Manufacturing Implementation	Current Challenges
**Geometry**	Honeycomb (hexagonal), lattice-like trabecular structures, gyroid surfaces (as found in corals), fractal branching (as seen in plants)	Lattice structures, gyroid geometries, fractal-inspired branching using 3D printing	Complexity in multiscale replication; geometric fidelity at micro/nanoscales
**Material Composition**	Hierarchical materials (e.g., bone, spider silk, seashells)	Gradient composites, metamaterials, bio-based polymers	Limited material versatility; lack of multifunctionality (e.g., self-healing, tunability, responsiveness)
**Functionality**	Adaptive systems (e.g., muscle contraction, self-healing skin)	4D printing with shape-memory polymers, stimuli-responsive hydrogels	Limited control over dynamic behavior; challenges in encoding functional responses
**Material Limitations**	Bio-composites evolved for strength, lightness, adaptability	Use of thermoplastics, resins, metals, experimental composites	Insufficient mimicry of biological versatility; scalability of advanced materials
**Printing Resolution**	Nano- to macroscale organization (e.g., lotus leaf, insect wings)	Micro-extrusion, two-photon polymerization, direct laser writing	Resolution constraints hinder replication of fine biological features
**Functional Integration**	Environmentally responsive and self-modifying biological systems	Embedded sensors, smart materials, hybrid structures	Difficulties in multifunctionality, stability, and long-term reliability

**Table 2:** Emerging frontiers in biomimetic additive manufacturing (BAM).

Approach	Biological Inspiration	Enabling Technology	Key Functionalities	Potential Applications
**4D Printing**	Plant tropisms, adaptive skin	Shape memory polymers, hydrogels, composites	Shape change over time, responsiveness to stimuli	Smart implants, responsive wearables, adaptive architecture
**Soft Robotics**	Octopus limbs, worm locomotion	Elastomers, pneumatic/hydraulic actuators	Flexible movement, dexterity, safe interaction	Minimally invasive surgery, soft grippers, disaster robotics
**Self-Morphing Structures**	Mimosa pudica, metamorphosis, seed dispersal	Shape memory alloys, programmable hydrogels	Complex transformations, self-assembly, deployability	Biomedical devices, aerospace structures, adaptive robotics

**Table 3:** Key challenges in biomimetic additive manufacturing (BAM) and current solutions.

Challenge Area	Description	Implications for BAM	Current Solutions/Research
**Material Science Bottlenecks**	Limited availability of bioequivalent smart materials that can mimic the dynamic, multifunctional properties of biological tissues.	Inability to replicate the full range of natural material properties (e.g., self-healing, adaptability, responsiveness). Affects functionality and real-world applications.	Research on bio-based polymers, hydrogels, shape-memory alloys, and bioinspired composites.
**Scalability Issues**	Difficulty in efficiently reproducing complex, large-scale biomimetic structures with high precision and consistency.	Loss of resolution, material inconsistencies, and structural integrity issues when scaling up 3D printing processes. Limits large-scale applications in industries like construction, healthcare, and aerospace.	Development of high-resolution 3D printing technologies, optimization of printing materials, and post-processing techniques.
**True Functional Mimicry vs. Esthetic Mimicry**	Esthetic mimicry focuses on replicating appearance, while true functional mimicry involves replicating natural systems’ full range of behaviors.	Esthetic mimicry fails to achieve the functional capabilities of natural systems, such as mechanical strength, flexibility, and adaptability. Limits the real-world performance of BAM products.	Interdisciplinary research combining material science, biology, and engineering to replicate natural functionalities.

**Table 4:** Future perspectives in biomimetic additive manufacturing (BAM).

Future Perspective	Description	Implications for BAM	Current Research/Trends
**Machine Learning and Generative Design**	Computational models and algorithms optimize designs based on biological principles.	Enables rapid iteration, material optimization, and performance prediction with minimal human input.	ML for predicting material behavior, generative algorithms for lattice and gyroid design.
**Living Materials and Hybrid Systems**	Integration of living cells with synthetic materials to enable responsive, self-healing behaviors.	Bridges biology and engineering, creating materials that evolve, adapt, or integrate with living systems.	Biohybrid printing, tissue scaffolds, plant-inspired architectures.
**Ethical, Ecological and Sustainability Considerations**	Environmental impact, biocompatibility, lifecycle assessment, and bioethics in design.	Promotes long-term viability, social acceptance, and regulatory compliance.	Green materials, ethical frameworks, circular manufacturing models.
**Standardization and Regulation**	Development of protocols and metrics for BAM validation and scalability.	Facilitates industrial adoption, ensures reproducibility, and enhances trust in BAM-based solutions.	ISO frameworks, biomimicry scoring, reproducibility studies.
**Interdisciplinary Collaboration**	Integration of expertise across biology, engineering, design, and computational science.	Essential for solving complex problems and translating research into real-world applications.	Joint research hubs, hybrid training programs, cross-disciplinary design platforms.

## Data Availability

No new data were created or analyzed in this study. Data sharing is not applicable to this article.

## Abbreviations

The following abbreviations are used in this manuscript:

**Abbreviation**	**Definition**
4D Printing	Additive manufacturing of dynamic, shape-changing structures over time
AI	Artificial Intelligence
AM	Additive Manufacturing
BAM	Biomimetic Additive Manufacturing
CAD	Computer-Aided Design
DNA	Deoxyribonucleic Acid
DOE	Design of Experiments
DLP	Digital Light Processing
ECM	Extracellular Matrix (often referenced in biohybrid systems)
FDM	Fused Deposition Modeling (a common 3D printing technique)
IoT	Internet of Things
ISO	International Organization for Standardization
LCEs	Liquid Crystal Elastomers (stimuli-responsive materials)
ML	Machine Learning
PLA	Polylactic Acid (common biopolymer used in 3D printing)
SLA	Stereolithography
SLS	Selective Laser Sintering
